# Prognostic significance of frailty in hospitalized elderly patients with community-acquired pneumonia: a retrospective cohort study

**DOI:** 10.1186/s12877-023-04029-3

**Published:** 2023-05-17

**Authors:** Hongye Zhao, Junlan Tu, Quan She, Min Li, Kai Wang, Weihong Zhao, Peng Huang, Bo Chen, Jianqing Wu

**Affiliations:** 1 Jiangsu Provincial Key Laboratory of Geriatrics, Department of Geriatrics, The First Affiliated Hospital of Nanjing Medical University, 300 Guangzhou Road, Jiangsu 210029 Nanjing, P.R. China; 2 Department of General Practice, The First People’s Hospital of Lianyungang, Lianyungang Clinical College of Nanjing Medical Unversity, Lianyungang, 222000 China; 3 Department of Epidemiology, Center for Global Health, School of Public Health, Nanjing Medical University, Nanjing, 211166 China

**Keywords:** community-acquired pneumonia, Frailty, FI-Lab, Elderly patients, Clinical outcome and prognosis

## Abstract

**Background:**

Frailty is associated with poor prognosis in a wide range of illnesses. However, its prognostic implications for older patients with community-acquired pneumonia (CAP) are not adequately addressed.

**Methods:**

In this study, patients were classified into 3 groups according to the frailty index based on standard laboratory tests (FI-Lab) score: robust (FI-Lab < 0.2), pre-frail (FI-Lab 0.2–0.35), and frail (FI-Lab ≥ 0.35). The relationships between frailty and all-cause mortality and short-term clinical outcomes (length of stay, duration of antibiotic therapy, in-hospital mortality) were examined.

**Results:**

Finally, 1164 patients were included, the median age was 75 years (interquartile range: 69, 82), and 438 patients (37.6%) were women. According to FI-Lab, 261(22.4%), 395(33.9%), and 508(43.6%) were robust, pre-frail, and frail. After adjustment for confounding variables, frailty was independently associated with prolonged antibiotic treatment (*p* = 0.037); pre-frailty and frailty were independently associated with longer inpatient days (*p* < 0.05 for both). The risk of in-hospital mortality was independently increased in frail patients (HR = 5.01, 95% CI = 1.51–16.57, *p* = 0.008) but not pre-frail patients (HR = 2.87, 95% CI = 0.86–9.63, *p* = 0.088) compared to robust patients. During a median follow-up of 33.9 months (interquartile range: 32.8 to 35.1 months), 408 (35.1%) patients died, of whom 29 (7.1%) were robust, 112 (27.5%) were pre-frail, and 267 (65.9%) were frail. Compared to robust patients, frail and pre-frail were significantly associated with increased risk for all-cause death (HR = 4.29, 95%CI: 1.78–10.35 and HR = 2.42 95%CI: 1.01–5.82, respectively).

**Conclusions:**

Frailty is common among older patients with CAP and is strongly associated with increased mortality, longer length of stay, and duration of antibiotics. A routine frail assessment at the admission of elderly patients with CAP is necessary as the first step for appropriate multidisciplinary interventions.

**Supplementary Information:**

The online version contains supplementary material available at 10.1186/s12877-023-04029-3.

## Introduction

Community-acquired pneumonia (CAP) is the most prevalent infectious disease in older adults and is associated with poor prognosis and high medical costs worldwide [1, 2]. CAP-associated morbidity and mortality increase with age. Despite medical advances, mortality in elderly patients with CAP is still high [3]. Identifying high-risk patients based on dynamic clinical indicators is essential to perform interventions on these variables to reduce the risk.

Frailty, a geriatric syndrome, is an age-related disease characterized by a decline in functions across multiple physiological systems and an increased vulnerability to stressors. This increases the risk of adverse clinical outcomes, including falls, hospitalization, and death. Frailty is also associated with advanced age, disabilities, and comorbidities [4, 5]. Frailty can be evaluated using various tools, while it currently lacks a universally accepted standard definition [6]. The frailty index (FI) is one of the most used tools for defining frailty and predicting patient death [7]. The frailty index based on laboratory test (FI-lab), defined as the proportion of aberrant results from the total of measured tests, is one method to assess frailty [8, 9]. With readily available laboratory test data in electronic medical records, frailty by the FI-lab might be evaluated objectively and uniformly. In elderly patients, a higher FI-Lab has been linked to death, readmissions, and longer hospital stays [10–12].

Previous research demonstrated that frailty provides additional value to disease-specific severity measures predicting mortality following acute medical hospitalizations [13, 14]. In older persons, frailty influenced the susceptibility and severity of pneumonia [15]. Compared to other clinical factors, frailty has the advantage that it is a modifiable risk factor on which physicians could act. However, the relationship between frailty and clinical events in older patients with CAP has not been adequately addressed.

In this study, we aim to evaluate the association of FI-Lab, created at admission with routine laboratory tests, with short-term outcomes (i.e., length of stay, duration of antibiotic therapy) and long-term outcomes (i.e., all-cause mortality) in a contemporary cohort of elderly patients with CAP.

## Methods

### Study population

This is a single-center retrospective observational cohort study, which initially included 1359 patients aged ≥ 65 years with a principal diagnosis of CAP admitted consecutively between January 2018 and December 2019 to the First Affiliated Hospital of Nanjing Medical University (NJMU). The diagnosis of CAP was established according to the Chinese Guidelines for Adult CAP (2016 Edition) [16]. The exclusion criteria were patients without medical records or routine laboratory variables (here, routine blood tests, blood biochemistry, and coagulation tests) or readmission or patients with missing data on follow-up. The research was approved by the Ethics Committee of the First Affiliated Hospital of NJMU, and the ethics number is 2020-SR-072.

### Data collection

Data were independently collected from electronic medical records by two researchers and cross-checked. Included indicators were as follows: age; height; weight; sex; smoking and drinking history; severity of CAP as measured by the CURB-65; comorbidities as measured by the age-adjusted Charlson Comorbidity Index (aCCI); laboratory results from the first 24 h of admission, routine blood tests, blood biochemistry, and coagulation tests, high sensitivity C-reactive protein, eGFR, procalcitonin; length of stay during hospitalization; duration of antibiotic therapy.

### Frailty assessment

Frailty was outlined by FI-Lab for this investigation. Following prior investigations, we constructed FI-Lab based on 44 laboratory variables evaluated from a fasting blood sample, including routine blood tests, blood biochemistry tests, and coagulation tests [8, 17] (Supplementary Table S1). Consistent with a previous study, we required at least 70% or 31 of the 44 variables to generate a valid FI-Lab score [9]. The cut-off points of the FI-Lab were robust/non-frail (< 0.2), pre-frail (0.2–0.35), and frail (≥ 0.35), according to earlier investigations [18, 19]. Furthermore, we also analyzed the FI-Lab score as a continuous variable.

### Endpoints and follow-up

The primary endpoint was all-cause mortality. The secondary endpoint was the length of hospital stay, antibiotic therapy duration and in-hospital mortality. Patients were followed up since the date of CAP admission. We obtained the information from electronic medical records, and conducted telephone interviews with patients for whom lacked system information, and the death information includes the status of survival and the time of death until January 1, 2022.

### Statistical analysis

Continuous data are expressed as a median with an interquartile range (25th to 75th percentiles), while categorical data are expressed as n (%). One-way ANOVA were performed to evaluate differences among groups. The chi-square test was used to compare proportions among groups. FI-Lab scores were evaluated as a categorical variable (robust, pre-frailty and frailty). Multiple linear regression was performed to assess the associations of FI-Lab with the length of hospital stay and duration of antibiotic therapy. Univariable Cox proportional regression evaluated the association between FI-Lab (categorical and continuous) and plausible confounding variables with in-hospital mortality and all-cause mortality. The variables based on a threshold *p*-value < 0.05 in the univariable analysis were considered in the multivariable Cox proportional regression analysis and a stepwise method was used. Time-to-event data are presented graphically using Kaplan–Meier curves. Log-rank tests were used to compare survival between groups. The binary Logistic regression model was used to predict the probability of death. The predictive accuracy of the indexes was validated using receiver operating characteristic (ROC) and quantified by the area under the curve (AUC) and 95% CIs. The SPSS 22.0, R version 4.1.2, and Prism 8 were applied for all statistical analyses and plots. The statistical significance was set as two-tailed with p at < 0.05.

## Results

### Patient characteristics

**Fig. 1:**
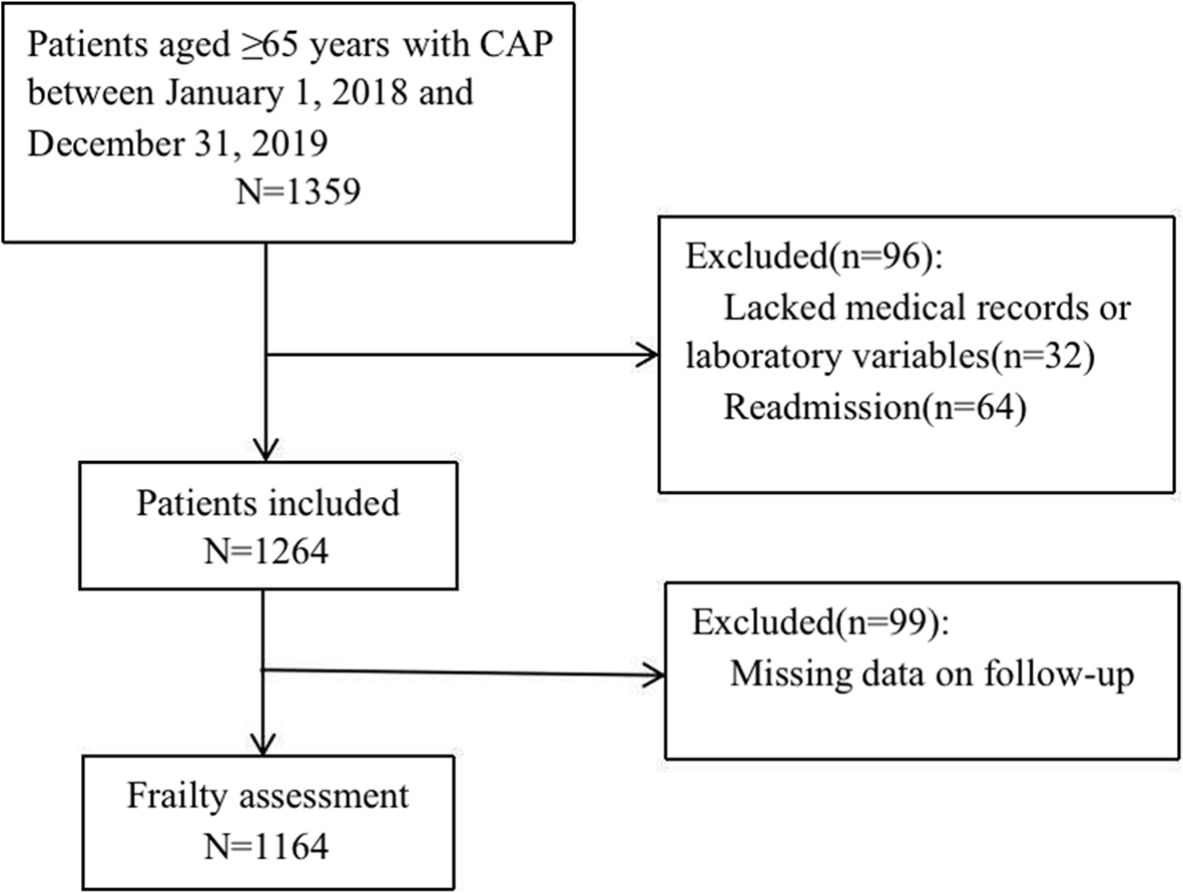
Study profile including selection

A cohort of 1164 older patients was finally included in this study by enrollment and exclusion criteria (Fig. 1). Among the patients, frail and pre-frail patients were older than robust patients (median age 77.0 vs. 77.0 vs. 71.0, *p* < 0.001). Robust patients had more female than pre-frail and frail groups (52.1% vs. 39.0% vs. 29.1%, *p* < 0.001). The inflammation-related indicators hs-CRP and PCT were significantly higher in the frail group than in the pre-frail or robust group (67.6 vs. 30.0 vs. 5.0, *p* < 0.001, 0.39 vs. 0.10 vs. 0.08, *p* < 0.001). Patients in the frail and pre-frail groups suffered more number of chronic diseases (aCCI score, 6 vs. 5 vs. 4, *p* < 0.001) and a higher burden of disease (CURB-65 score, 2 vs. 1 vs. 1, *p* < 0.001) than in the robust group. Smoking and drinking history did not differ significantly within groups (Table 1).

**Table 1:** Characteristics of the study population according to frailty assessed by FI-Lab

Variables	Robust(*n* = 261)	Pre-frailty(*n* = 395)	Frailty(*n* = 508)	*P*
Age(years)	71.0(67.0,76.5)	77.0(70.0,83.0)	77.0(71.0,84.0)	< 0.001
Female,n(%)	136(52.1)	154(39.0)	148(29.1)	< 0.001
BMI(kg/m2)	24.2(21.8,26.0)	22.78(19.9,25.1)	22.04(19.8,24.8)	< 0.001
Smoking history,n(%)				0.713
No	194(74.3)	294(74.4)	367(72.2)	
Yes	67(25.7)	101(25.6)	141(27.8)	
Drinking history,n(%)				0.468
No	222(85.1)	348(88.1)	436(85.8)	
Yes	39(14.7)	47(11.9)	72(14.2)	
WBC(× 10^9/L)	5.8(4.9,7.2)	7.0(5.3,8.5)	8.6(5.9,11.9)	< 0.001
LYMPH(× 10^9/L)	1.5(1.2,1.9)	1.1(0.8,1.6)	0.9(0.6,1.2)	< 0.001
NEUT(× 10^9/L)	3.6(2.8,4.7)	4.9(3.5,6.4)	6.8(4.2,10.0)	< 0.001
eGFR(ml/min)	84.0(72.0,91.0)	83.0(65.0,91.0)	72.0(30.0,91.0)	< 0.001
hs-CRP(mg/L)	5.0(3.1,19.7)	30.0(7.3,59.9)	67.6(31.7,101.0)	< 0.001
PCT(ng/ml)	0.08(0.04,0.10)	0.10(0.05,0.21)	0.39(0.13,1.44)	< 0.001
aCCI	4(3,5)	5(4,6)	6(4,8)	< 0.001
CURB-65	1(1,1)	1(1,2)	2(1,2)	< 0.001
LOS(days)	8.0(6.0,11.0)	11.0(8.0,15.0)	12.0(8.0,18.0)	< 0.001
Duration of antibiotic therapy	7.0(5.0,9.0)	9.0(7.0,13.0)	11.0(7.0,15.0)	< 0.001
Death,n(%)	29(11.1)	112(28.4)	267(52.6)	< 0.001

Abbreviations: *BMI* Body mass index, *WBC* White blood cell, *NEUT* Neutrophil, *LYMPH* Lymphocyte, *eGFR* estimated glomerular filtration rate, *hs-CRP* high sensitivity C-reactive protein, *PCT* Procalcitonin, *CURB-65* Confusion, blood Urea nitrogen, Respiratory rate, systolic or diastolic Blood pressure, and Age > 65, *LOS* Length of stay during hospitalization

### Frailty and short-term outcomes

We observed a longer duration of antibiotic therapy and increased hospital stay among frail and pre-frail patients compared to robust patients (11.0 vs. 9.0 vs. 7.0 days, *p* < 0.001, 12.0 vs. 11.0 vs. 8.0 days, *p* < 0.001; Table 1).

In the multiple linear regression analysis, after adjustment for confounding variables, frailty was independently associated with prolonged antibiotic treatment (*p* = 0.037; Supplementary Table S2), while pre-frail and frail groups were independently associated with longer inpatient days (*p* < 0.05 for both; Supplementary Table S3). As continuous variables, the FI-Lab score was independently associated with a longer duration of antibiotic therapy and increased length of hospital stay after adjusting for confounding factors (*p* = 0.021, Supplementary Table S2; *p* < 0.001; Supplementary Table S3).

The risk of in-hospital mortality was dramatically and independently increased in frail patients (HR = 5.01, 95% CI = 1.51–16.57, *p* = 0.008) but not pre-frail patients (HR = 2.87, 95% CI = 0.86–9.63, *p* = 0.088) compared to robust patients(Supplementary Table S4).

### Mortality according to FI-Lab

During a median follow-up of 33.9 months (interquartile range: 32.8 to 35.1 months), 408 (35.1%) patients died (Table 1). The risk of all-cause mortality was dramatically increased in frail patients (HR = 6.81, 95% CI = 4.64–10.00, *p* < 0.001) and pre-frail patients (HR = 2.86, 95% CI = 1.90–4.30, *p* < 0.001) compared to robust patients. The prognostic role of frailty and pre-frailty for all-cause mortality were still significant after adjusting for potential confounding variables (HR = 3.61, 95%CI = 2.41–5.40, *p* < 0.001; HR = 2.02, 95% CI = 1.34–3.05, *p* = 0.001; respectively, Table 2). Equally, as continuous variables, after adjusting for confounding factors, the increment of the FI-Lab score was associated with an increased risk of mortality (adjusted HR per 0.10 increment in score 1.58, 95% CI 1.36–1.83; Table 3).

**Table 2:** Risk factors associated with death(FI-Lab as categorical variable)

variable	Univariable HR (95% CI)	*p* value	Multivariable HR (95% CI)	*p* value
Age(years)	1.01(1.00–1.21)	0.116		
Male Sex(vs female)	0.97(0.88–1.07)	0.532		
BMI(kg/m2)	0.80(0.78–0.83)	< 0.001	0.85(0.82–0.88)	< 0.001
Smoker (vs nonsmoker)	0.90(0.72–1.12)	0.341		
Drinking (vs no)	1.24(1.05–1.46)	0.012		
WBC(× 10^9/L)	1.07(1.05–1.09)	< 0.001		
LYMPH(× 10^9/L)	0.55(0.46–0.67)	< 0.001		
NEUT(× 10^9/L)	1.09(1.07–1.11)	< 0.001	1.05(1.02–1.07)	< 0.001
eGFR(ml/min)	0.99(0.99–0.99)	< 0.001	1.01(1.00–1.01)	< 0.001
CRP(mg/L)	1.01(1.00–1.01)	< 0.001		
PCT(ng/ml)	1.03(1.02–1.04)	< 0.001		
aCCI	1.30(1.26–1.35)	< 0.001	1.17(1.12–1.23)	< 0.001
CURB-65	2.23(1.98–2.53)	< 0.001	1.51(1.30–1.75)	< 0.001
FI-Lab, categorical variable				
Robust	1		1	
Pre-frail	2.86(1.90–4.30)	< 0.001	2.02(1.35–3.05)	0.001
Frail	6.81(4.64–10.00)	< 0.001	3.61(2.41–5.40)	< 0.001

***Abbreviations**** BMI* Body mass index, *WBC* White blood cell, *NEUT* Neutrophil, *LYMPH* Lymphocyte, *eGFR* estimated glomerular filtration rate, *hs-CRP* high sensitivity C-reactive protein, *PCT* Procalcitonin, *CURB-65* Confusion, blood Urea nitrogen, Respiratory rate, systolic or diastolic Blood pressure, and Age > 65, *LOS* Length of stay during hospitalization, *HR* hazard ratio, *OR* Odds ratio

**Table 3:** Risk factors associated with death(FI-Lab as continuous variables)

variable	Univariable HR (95% CI)	*p* value	Multivariable HR (95% CI)	*p* value
Age(years)	1.07(1.06–1.08)	< 0.001	1.04(1.02–1.06)	< 0.001
Male Sex(vs female)	1.49(1.20–1.83)	< 0.001	1.03(0.71–1.48)	0.877
BMI(kg/m2)	0.94(0.91–0.97)	< 0.001	0.95(0.91–0.99)	0.011
Smoker (vs nonsmoker)	0.90(0.71–1.12)	0.337		
Drinking (vs no)	0.65(0.47–0.91)	0.012	0.55(0.29–1.04)	0.067
WBC(× 10^9/L)	1.07(1.06–1.09)	< 0.001	0.61(0.40–0.93)	0.022
LYMPH(× 10^9/L)	0.55(0.45–0.66)	< 0.001	1.97(1.07–3.63)	0.009
NEUT(× 10^9/L)	1.09(1.07–1.11)	< 0.001	1.72(1.11–2.65)	0.014
eGFR(ml/min)	0.99(0.98–0.99)	< 0.001	1.01(1.00–1.01)	0.084
CRP(mg/L)	1.01(1.00–1.01)	< 0.001	1.00(0.99–1.00)	0.134
PCT(ng/ml)	1.03(1.02–1.04)	< 0.001	1.00(0.97–1.01)	0.248
aCCI	1.31(1.26–1.35)	< 0.001	1.13(1.05–1.21)	< 0.001
CURB-65	2.25(1.99–2.54)	< 0.001	1.29(1.01–1.64)	0.043
FI-Lab, continuous variables^a^	1.89(1.73–2.01)	< 0.001	1.58(1.36–1.83)	< 0.001

Abbreviations *BMI* Body mass index, *WBC* White blood cell, *NEUT* Neutrophil, *LYMPH* Lymphocyte, *eGFR* estimated glomerular filtration rate, *hs-CRP* high sensitivity C-reactive protein, *PCT* Procalcitonin, *CURB-65* Confusion, blood Urea nitrogen, Respiratory rate, systolic or diastolic Blood pressure, and Age > 65, *LOS* length of stay during hospitalization

^a^A change in FI-Laboratory represents a 0.10 increase

**Fig. 2:**
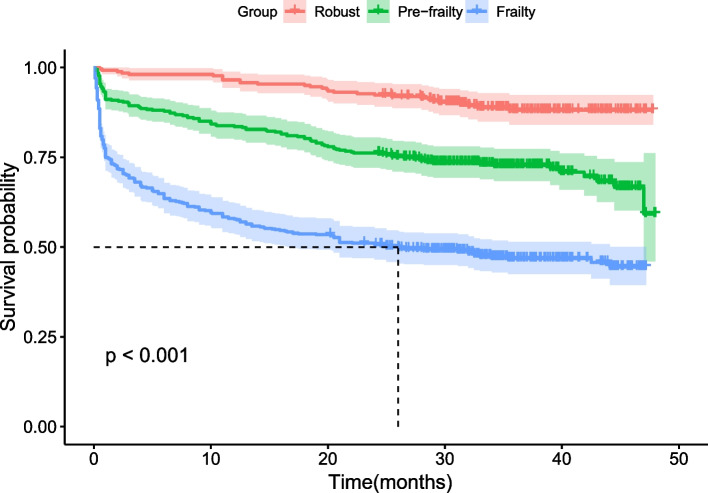
Kaplan–Meier analysis plot for all-cause mortality according to baseline frailty status assessed by FI-Lab scores

Figure 2 shows the survival curves of the study population according to their FI-Lab-based frailty status, and the curves differ significantly among the three groups (log-rank *p* < 0.001).

**Fig. 3:**
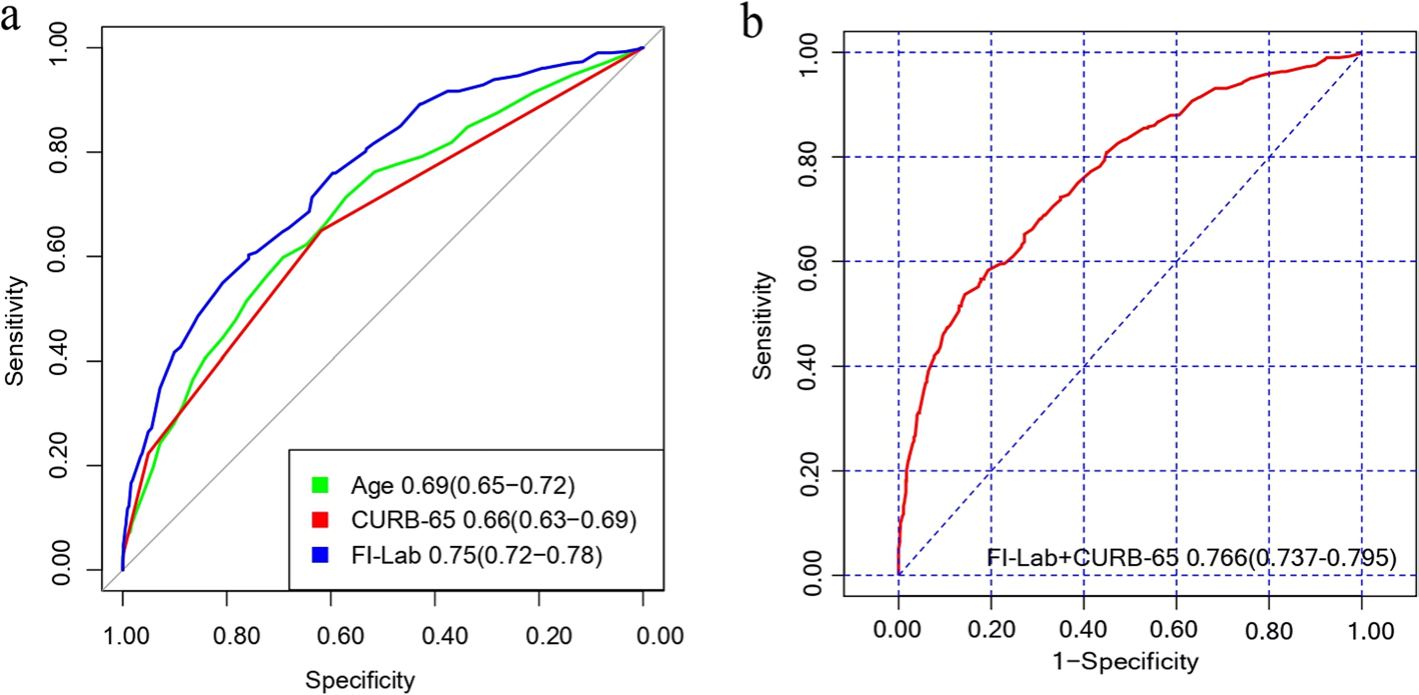
The ROC curve indicated possible predictive accuracy of FI-Lab on mortality of patients with CAP. **a** ROC curves for age, CURB-65, FI-Lab. **b** ROC curve for CURB-65 + FI-Lab

Given that the CURB-65 score is an index of severity for pneumonia, we compared the FI-Lab to CURB-65 for the prediction of death after CAP hospitalization. The FI-Lab score outperformed the CURB-65 at predicting all-cause mortality, as was seen by the ROC curve (AUC 0.75 vs. 0.66, *p* < 0.001; Fig. 3a). For mortality risk prediction, the FI-Lab score provided a significant incremental prognostic value on the CURB-65 score (AUC 0.77 vs. 0.66, *p* < 0.001; Fig. 3b).

## Discussion

As defined by FI-Lab, frailty is common in older patients with CAP and is associated with a poor prognosis regardless of the CURB-65, aCCI, and other risk factors. With FI-Lab-based frailty provided a significant increase in the statistical accuracy of the CURB-65, frailty may be a critical, potentially modifiable risk and therapeutic target in older patients with CAP.

The first issue that should be evaluated is the prevalence of frailty. Based on the FI-Lab scores, 47.3% of our study’s elderly patients with CAP were labeled as frailty, and 33.9% were pre-frailty. Few studies have analyzed the prevalence of frailty in older patients with CAP. Luo et al. [20] reported that 66.8% of elderly patients with CAP were frail, using the Fried frailty phenotype in a cohort of 256 patients ages ≥ 65 years. However, clinical physicians are not aware of the prevalence of frailty. Despite its increasing importance, frailty is often not recognized and untreated. Accordingly, the first lesson that can be drawn from our study is the need to screen for frailty in older patients admitted for CAP. The five most commonly used methods for the assessment of frailty were CSF, FI, frailty phenotype, frail scale, EFS, and FI-lab was one of FI [21]. The FI-Lab scores analyzed are simple to calculate and useful to identify patients at risk for frailty. Frailty was more prevalent in men in our study, which is consistent with previous studies [8, 22]. However, most studies reported that women were more likely to be frail than men, even though women typically live longer [23, 24]. This phenomenon might be attributed partly to the frailty assessments being based on comprehensive geriatric assessments, which may not include all factors affecting life expectancy in older people [23]. In addition, we found that frail patients had significantly older age, greater hs-CRP and PCT, more comorbidities, and higher CURB-65 scores.

The second point to highlight is the relationship between FI-Lab-based frailty and short-term outcomes, including prolonged antibiotic treatment duration, longer length of stay and in-hospital death. We found that, regardless of clinical variables, frailty was still associated with adverse clinical events. This is consistent with Ellis et al. [11], who reported that higher CFS and FI-Lab scores were associated with more days in hospital. The FI-Lab can assist in identifying complex older adults at hospital admission who have accumulated multiple health deficits and are at an increased risk of adverse outcomes.

The third aspect of remarking is the relationship between frailty and all-cause mortality. Investigations have shown that high and moderate FI-LAB scores were associated with worse in-hospital and post-hospital outcomes, and FI-LAB can stratify older adults at increased risk of death alone [25], we proved this. According to a study using a large general older population hospitalized for pneumonia, frailty, measured by the Hospital Frailty Risk Score, was associated with mortality [26]. A strong relation between mortality and FI-Lab-based frailty was discovered in another investigation among acutely admitted older medical patients [12]. We found that, regardless of CURB-65, aCCI, and other risk variables, death in older CAP patients was independently influenced by FI-Lab-based frailty. CURB-65 scores were used for patients with pneumonia to predict mortality. Consistent with a prior study [13], we found that the FI-Lab combined with CURB-65 largely improved death prediction compared with CURB-65 alone. Several factors may explain the relationship in our study. Firstly, frailty represents the accumulation of cellular, tissue, and organ deficits damaged at those levels but not removed or repaired [9, 27]. The highest FI-Lab score with the significantly decreased survival point to the lethality of clinical deficits. Secondly, FI-Lab represents a more accurate and objective assessment than CGA-based frailty indices when predicting patient mortality [8, 9, 28].

All these findings strongly support the need for physicians to integrate in their daily practice the identification of frailty. Screening older patients with CAP for frailty might identify patients at high risk of adverse clinical outcomes, which might benefit from secondary prevention programs to improve their prognosis. Various multidisciplinary strategies have been developed to help prevent and treat frailty, including lifestyle changes, physical activities, and nutritional support [29, 30]. Clinicians should stay abreast of the most recent scientific evidence to provide helpful and practical patient guidance. The intervention should begin during CAP hospitalization because of the patient’s greater vulnerability to change in that period and also continue even after discharge. Meanwhile, preventing frailty to avoid elderly patients with CAP from experiencing a decline in their functional status and general health may be even more critical. Well-designed and well-executed future studies are needed to arrive at a reasonable evaluation of the impact of an intervention on frailty and CAP.

Our study has several limitations. First, this is a single-center, retrospective cohort study with a limited sample size and inevitable selection bias. Second, we did not compare the prognostic value of FI-Lab with other comprehensive frailty assessments, such as the physical frailty phenotype and the FI of accumulative deficits complex. This is relevant because frailty is a complicated issue, especially in older adults, because of various etiologies and determinants. Confirmation of our findings by more researchers and in other countries with various health care and social systems would be welcome.

## Conclusions

Frailty assessed by routine laboratory data could allow clinicians to identify older patients with CAP at elevated risk for in-hospital and all-cause mortality, a longer hospital stay, and prolonged duration of antibiotics. Adequate assessment of frailty status can help improve the prognosis of elderly patients with CAP, selecting those who may benefit from support. This information can be translated into future trials to optimize the outcomes of older patients with CAP who are frail or pre-frail.

## Supplementary Information

**Additional file 1.** **Supplementary Table S1.** Laboratory variables for frailty index.** Supplementary Table S2.** Association of frailty and duration of antibiotic therapy by multiple liner regression analyses.** Supplementary Table S3.** Association of frailty and length of stay by multiple liner regression analyses. **Supplementary Table S4.** Risk factors associated with in-hospital death (FI-Lab as categorical variable).

## Data Availability

The datasets used and/or analysed during the current study available from the corresponding author on reasonable request.
