# Supplementary material for: Prognostic significance of frailty in hospitalized elderly patients with community-acquired pneumonia: a retrospective cohort study
Source: BMC Geriatr. 2023 May 17;23:308. doi: 10.1186/s12877-023-04029-3 (PMC10193599; doi:10.1186/s12877-023-04029-3)
Supplement: Supplementary file 1 — Additional file 1. Supplementary Table S1. Laboratory variables for frailty index. Supplementary Table S2. Association of frailty and duration of antibiotic therapy by multiple liner regression analyses. Supplementary Table S3. Association of frailty and length of stay by multiple liner regression analyses. Supplementary Table S4. Risk factors associated with in-hospital death (FI-Lab as categorical variable). [file 12877_2023_4029_MOESM1_ESM.docx]

**Supplementary Table S1** Laboratory variables for frailty index

|  | Item | Reference range |
| --- | --- | --- |
| Routine blood tests | | |
| 1 | WBC(10^9/L) | 3.5-9.5 |
| 2 | LYMPH(10^9/L) | 1.10-3.20 |
| 3 | MONO(10^9/L) | 0.10-0.60 |
| 4 | NEUT(10^9/L) | 1.8-6.3 |
| 5 | EO(10^9/L) | 0.02-0.52 |
| 6 | BASO(10^9/L) | 0.00-0.06 |
| 7 | RBC(10^12/L) | 3.80-5.10(F)  4.30-5.80(M) |
| 8 | Hb(g/L) | 115-150(F)  130-175(M) |
| 9 | HCT(%) | 35.0-45.0(F)  40.0-50.0(M) |
| 10 | MCV(fL) | 82.0-100.0 |
| 11 | MCH(pg) | 27.0-34.0 |
| 12 | MCHC(g/L) | 316-354 |
| 13 | RDW-CV(%) | 10.00-15.70 |
| 14 | RDW-SD(fL) | 37.00-54.00 |
| 15 | PLT(10^9/L) | 125-350 |
| Blood biochemistry | | |
| 16 | ALT(U/L) | 7.0-40.0(F)  9.0-50.0(M) |
| 17 | AST(U/L) | 13.0-35.0(F)  15.0-40.0(M) |
| 18 | ALP(U/L) | 30-120 |
| 19 | GGT(U/L) | 7.0-45.0(F)  10.0-60.0(M) |
| 20 | LDH(U/L)） | 140-271 |
| 21 | CK(U/L) | 0-145(F)  0-171(M) |
| 22 | TBIL(umol/L) | 6.0-22.0 |
| 23 | DBIL(umol/L) | 0.0-6.8 |
| 24 | IDBIL(umol/L) | 0.0-20.0 |
| 25 | TC(mmol/L) | 3.00-5.70 |
| 26 | TG(mmol/L) | 0.00-2.25 |
| 27 | HDL-C(mmol/L) | 1.03-1.55 |
| 28 | LDL-C(mmol/L) | 2.60-4.10 |
| 29 | TP(g/L) | 65.0-85.0 |
| 30 | ALB(g/L) | 40.0-55.0 |
| 31 | GLU(mmol/L) | 3.90-6.10 |
| 32 | UREA(mmol/L) | 2.90-8.20 |
| 33 | CREA(umol/L) | 44.0-133.0 |
| 34 | URIC(umol/L) | 155-357(F)  208-428(M) |
| 35 | CA(umol/L) | 2.20-2.65 |
| 36 | PHOS(mmol/L) | 0.81-1.45 |
| 37 | MG(mmol/L) | 0.77-1.03(F)  0.73-1.06(M) |
| 38 | K(mmol/L) | 3.5-5.3 |
| 39 | NA(mmol/L) | 137.0-147.0 |
| 40 | CL(mmol/L) | 99.0-110.0 |
| Blood coagulation | | |
| 41 | PT (s) | 8.0-14.0 |
| 42 | APTT(s) | 25.0-31.3 |
| 43 | FIB(g/L) | 2.0–4.0 |
| 44 | INR | 0.88–1.15 |

**Supplementary Table S2** Association of frailty and duration of antibiotic therapy by multiple liner regression analyses.

|  | R^2^=0.13, Adjusted R^2^ 0.12  p<0.0001, F=12.45 | | R^2^=0.12, Adjusted R^2^ 0.11  p<0.0001, F=14.01 | |
| --- | --- | --- | --- | --- |
| variable | β (95%CI) | p value | β (95%CI) | p value |
| Age(years) | 0.14(0.07,0.21) | <0.001 | 0.13(0.062,0.20) | <0.001 |
| Male Sex(vs female) | 1.51(0.20,2.82) | 0.024 | 1.58(0.27,2.89) | 0.018 |
| smoker(vs nonsmoker) | -2.22(-3.65,-0.79) | 0.002 | -2.21(-3.64,-0.78) | 0.003 |
| hsCRP(mg/L) | 0.02(0.00,0.02) | 0.007 | 0.02(0.00,0.03) | 0.008 |
| PCT(ng/ml) | 0.16(0.07,0.27) | 0.001 | 0.16(0.06,0.26) | 0.001 |
| FI-Lab, categorical variable |  |  |  |  |
| Robust |  |  |  |  |
| Pre-frailty | 0.22(-1.60,2.03) | 0.814 |  |  |
| Frailty | 1.96(0.12,3.80) | 0.037 |  |  |
| FI-Lab, continuous variables^＊^ |  |  | 0.57(0.09,1.05) | 0.021 |

Note:^＊^A change in FI-Laboratory represents a 0.10 increase.

Abbreviations: hs-CRP, high sensitivity C-reactive protein; PCT, Procalcitonin.

**Supplementary Table S3** Association of frailty and length of stay by multiple liner regression analyses.

|  | R^2^=0.13, Adjusted R^2^ 0.13  p<0.0001, F=35.77 | | R^2^=0.13, Adjusted R^2^ 0.13  p<0.0001, F=43.32 | |
| --- | --- | --- | --- | --- |
| variable | β (95%CI) | p value | β (95%CI) | p value |
| Age(years) | 0.23(0.17,0.28) | <0.001 | 0.22(0.17,0.28) | <0.001 |
| Male Sex(vs female) | 1.59(0.60,2.58) | 0.002 | 1.62(0.62,2.61) | 0.00147 |
| smoker(vs nonsmoker) | -1.89(-2.97,-0.81) | 0.001 | -1.91(-2.99,-0.83) | 0.000551 |
| FI-Lab, categorical variable |  |  |  |  |
| Robust |  |  |  |  |
| Pre-frailty | 2.06(0.87,3.25) | 0.001 |  |  |
| Frailty | 3.73(2.58,4.88) | <0.001 |  |  |
| FI-Lab, continuous variables^＊^ |  |  | 0.94(0.63,1.25) | <0.001 |

Note:^＊^A change in FI-Laboratory represents a 0.10 increase.

**Supplementary Table S4** Risk factors associated with in-hospital death(FI-Lab as categorical variable)

| variable | Univariable HR  (95% CI) | p value | Multivariable  HR (95% CI) | p value |
| --- | --- | --- | --- | --- |
| Age(years) | 1.01(1.00-1.21) | 0.543 |  |  |
| Male Sex(vs female) | 1.01(0.84-1.22) | 0.917 |  |  |
| BMI（kg/m2） | 0.78(0.76-0.84) | <0.001 | 0.87(0.83-0.91) | <0.001 |
| Smoker (vs nonsmoker) | 0.91(0.60-1.38) | 0.654 |  |  |
| Drinking (vs no) | 1.08(0.81-1.42) | 0.614 |  |  |
| WBC（x10^9/L） | 1.09(1.06-1.11) | <0.001 |  |  |
| LYMPH(x10^9/L) | 0.48(0.33-0.69) | <0.001 |  |  |
| NEUT(x10^9/L) | 1.11(1.09-1.14) | <0.001 | 1.04(1.01-1.07) | 0.015 |
| eGFR（ml/min） | 0.99(0.98-1.00) | <0.001 | 1.02(1.01-1.02) | <0.001 |
| CRP（mg/L） | 1.01(1.01-1.01) | <0.001 | 1.00(1.00-1.01) | 0.028 |
| PCT（ng/ml） | 1.03(1.02-1.05) | <0.001 |  |  |
| aCCI | 1.34(1.26-1.42) | <0.001 | 1.20(1.17-1.30) | <0.001 |
| CURB-65 | 3.07(2.52-3.74) | <0.001 | 2.01(1.60-2.54) | <0.001 |
| FI-Lab, categorical variable |  |  |  |  |
| Robust | 1 |  | 1 |  |
| Pre-frail | 5.39(1.62-17.88) | 0.006 | 2.87(0.86-9.63) | 0.088 |
| Frail | 17.26(5.46-54.57) | <0.001 | 5.01(1.51-16.57) | 0.008 |
